# Cervical cancer detection by DNA methylation analysis in urine

**DOI:** 10.1038/s41598-019-39275-2

**Published:** 2019-02-28

**Authors:** Barbara C. Snoek, Annina P. van Splunter, Maaike C. G. Bleeker, Maartje C. van Ruiten, Daniëlle A. M. Heideman, W. Frederik Rurup, Wina Verlaat, Hans Schotman, Mignon van Gent, Nienke E. van Trommel, Renske D. M. Steenbergen

**Affiliations:** 1 Amsterdam UMC, Vrije Universiteit Amsterdam, Pathology, Cancer Center Amsterdam, De Boelelaan, 1117 Amsterdam, Netherlands; 2 BIOS Lab on a Chip group, MESA+ and MIRA institutes, University of Twente, Enschede, Netherlands; 3 Amsterdam UMC, Vrije Universiteit Amsterdam, Clinical Chemistry, De Boelelaan, 1117 Amsterdam, Netherlands; 4 Department of Gynecologic Oncology, Netherlands Cancer Institute - Antoni van Leeuwenhoek Hospital, Amsterdam, Netherlands

## Abstract

Urine samples provide a potential alternative to physician-taken or self-collected cervical samples for cervical screening. Screening by primary hrHPV testing requires additional risk assessment (so-called triage) of hrHPV-positive women. Molecular markers, such as DNA methylation, have proven most valuable for triage when applied to cervical specimens. This study was set out to compare hrHPV and DNA methylation results in paired urine and cervical scrapes, and to evaluate the feasibility of DNA methylation analysis in urine to detect cervical cancer. Urine samples (n = 41; native and sediment) and paired cervical scrapes (n = 38) from cervical cancer patients, and urine from 44 female controls, were tested for hrHPV and 6 methylation markers. Results on native urine and sediment were highly comparable. A strong agreement was found between hrHPV testing on urine and scrapes (kappa = 0.79). Also, methylation levels in urine were moderately to strongly correlated to those detected in scrapes (*r* = 0.508–0.717). All markers were significantly increased in urine from cervical cancer patients compared to controls and showed a good discriminatory power for cervical cancer (AUC = 0.744–0.887). Our results show a good agreement of urine-based molecular analysis with reference cervical samples, and suggest that urine-based DNA methylation testing may provide a promising strategy for cervical cancer detection.

## Introduction

Cervical cancer is the fourth most common cancer in women worldwide, affecting 528,000 women and leading to 266,000 deaths each year^1^. Virtually all cervical cancers are caused by a persistent, high-risk HPV (hrHPV) infection. The slow progression from premalignant Cervical Intraepithelial Neoplasia (CIN) to a malignancy provides ample time for early detection and necessary action. Current cervical screening programs use cytology or primary hrHPV testing which focus on detection of abnormal cells and presence of hrHPV infection, respectively. Both approaches, however, have limitations. Cytology is limited by the subjectivity of the analysis and relatively limited and variable sensitivity to detect cervical (pre)malignancies, which ranges from 50–80%^2^. HrHPV screening cannot differentiate between a transient productive infection and a persistent transforming infection, which lowers the specificity of the test. While a combined approach, either co-testing or cytology triage of HPV positives, mitigates a number of these concerns, the subjective nature and low sensitivity of cytology testing remains a relevant problem. This indicates that efforts towards a more objective triage strategy are needed. Recent studies from others and us have shown that testing of hrHPV-positive women for hypermethylated genes offers an objective triage tool for the detection of CIN3 and cervical cancer^3–7^.

Besides the use of the most optimal screening test, the success of any screening program also depends on attendance. Many women experience obtaining cervical scrapes as an unpleasant and invasive procedure^8,9^. As a considerable percentage of cervical cancer is diagnosed in the population of screening non-attendees^10^, there is need for an approach that will reach these women. Although self-sampling of cervico-vaginal specimens increases screening attendance^11^, recent studies focusing on hrHPV detection in urine have established that urine sampling is preferred over cervical sample collection either by physician or by self-sampling^9,12^. The use of urine for screening has many additional benefits. It offers an easily manageable manner of sampling, with the potential of large-scale application^13,14^.

Given the need for triage testing in primary hrHPV-based screening, previous studies of us have focused on DNA methylation analysis in cervical scrapes and self-samples^3,4^. Furthermore, a number of studies have reported on the use of DNA methylation testing in urine for the detection of other cancer types, such as bladder (reviewed by^15^) and prostate cancer^16,17^. These studies, together with our encouraging data on DNA methylation analysis in cervical scrapes and self-samples, indicate that DNA methylation testing in urine could provide a promising noninvasive method for cervical cancer detection.

This study was set out to assess the feasibility of DNA methylation analysis in urine using 6 previously identified DNA methylation markers^18,19^. We collected urine and cervical scrapes from women with cervical cancer and first tested which urine component, sediment or unfractioned native urine, is most suitable for hrHPV detection and DNA methylation analysis. Secondly, we compared hrHPV and DNA methylation results in paired urine and cervical scrapes and evaluated the ability of urine-based DNA methylation testing for cervical cancer detection.

## Methods

### Sample collection and processing

Urine samples (n = 43) and paired cervical scrapes (n = 38) were collected from cervical cancer patients, aged 27 to 86 at the Department of Gynecology, the Netherlands Cancer Institute, Amsterdam The Netherlands between February 2016 and March 2017. Voided urine samples were provided by participating women prior to surgery and collected in containers. Urine samples were processed within 24 hours. Surgical pathology reports were verified to confirm presence of cervical cancer at time of sample collection. Tumor stage and age are indicated in Table 1. Informed written consent was obtained from all patients and research protocols were approved by the Medical Ethics Committee of the Netherlands Cancer Institute.

**Table 1:** HrHPV test results in urine samples.

No.	Cancer type	Stage	Age	Native urine	Urine sediment	Cervical scrape
1^	SCC	IIB	52	NA**	positive (other)	positive (other)
2^	SCC	IIB	59	NA**	positive (16)	NA***
3^	SCC	IIB	51	positive (other)	positive (other)	positive (other)*
4^	AC	IBII	43	positive (16)	positive (16)	positive (16)*
5	SCC	IBI	31	positive (18)	positive (18)	positive (18)
6	SCC	IIB	56	positive (other)	positive (other)	positive (other)
7	AC	IBI	52	negative	negative	negative
8^	SCC	IIB	82	positive (16)	positive (16)	NA***
9	SCC	IBI	43	positive (16)	positive (16)	positive (16)
10	SCC	IIB	83	positive (other)	positive (other)	positive (other)
11	SCC	IBI	43	positive (16)	positive (16)	positive (16)
12	ASC	IBI	32	positive (16, 18)	positive (16, 18)	positive (16, 18)
13	ASC	IBI	34	positive (18)	positive (18)	positive (18)
14	SCC	IIB	36	positive (18)	positive (18)	positive (18)
15	AC	IBI	41	positive (16)	positive (16)	positive (16)
16	SCC	IBII	29	positive (other)	positive (other)	positive (other)
17	SCC	IIAI	75	positive (16)	positive (16)	positive (16)
18	SCC	IIIB	86	positive (16)	positive (16)	positive (16)
19	SCC	IIB	51	positive (16)	positive (16)	positive (16)
20	SCC	IIB	43	positive (16)	positive (16)	positive (16)
21	SCC	IIB	82	positive (16)	positive (16)	positive (16)
22	SCC	IIB	34	positive (other)	positive (other)	positive (other)
23	SCC	IBI	69	negative	negative	positive (other)
24	SCC	IBI	66	positive (16)	positive (16)	positive (16)
25	SCC	IBI	51	negative	positive (16)	positive (16)
26	SCC	IIB	76	negative	positive (other)	positive (other)
27	SCC	IIB	50	positive (16)	positive (16)	positive (16)
28	AC	IBI	46	positive (16)	positive (16)	positive (16)
29	SCC	IIIB	72	positive (other)	positive (other)	positive (other)
30^	SCC	IVB	35	positive (16)	positive (16)*	positive (16, other)
31	SCC	IIB	55	positive (18)	negative	positive (18)
32	SCC	IVB	80		positive (16)	positive (16)
33	SCC	IIAI	55		positive (16)	positive (16)
34	SCC	IBI	35		negative	negative
35	AC	IBII	45		positive (16)	positive (16)
36	SCC	IIIB	50		positive (18)	positive (18)
37	AC	IBI	27		positive (16, other)	positive (16, other)
38	AC	IIAI	82		negative	negative
39	SCC	IBI	31		positive (16)	positive (16)
40	SCC	IIB	46		positive (other)	positive (other)
41	AC	IBI	62		positive (18)	positive (18)
42	SCC	IIB	59		negative	negative
43	SCC	IBI	47		negative	positive (other)

Native urine (n = 31), urine sediments (n = 43), and cervical scrapes (n = 43) from women with cervical cancer were included. Urine component analysis (sediment versus native urine) was performed on paired urine sediment and native urine. Excluded samples are indicated by ^; three samples were excluded based on low quality (indicated by*), for two patients there was insufficient amount of DNA from native urine (indicated by**), and two patients did not obtain a cervical scrape (indicated by***). This resulted in a total set of 28 samples for urine component analysis (upper part) and 38 samples for comparison between urine sediment and cervical scrapes (complete set). Abbreviations: SCC, squamous cell carcinoma; AC, adenocarcinoma; ASC, adenosquamous carcinoma; NA, not available; ND, not done.

Control samples (n = 47; all females aged 30 to 60) originated from women without known malignancy of which urine was collected for routine diagnostic purposes. These samples were supplied by the Department of Clinical Chemistry of the VU University Medical Center, Amsterdam, and were used anonymously in accordance with the code of conduct for responsible use^20^. Accordingly, no data was available on prior HPV and disease status.

Urine samples were processed within 24 hours. In order to evaluate different fractions of the urine, (1) 15 mL of urine was centrifuged at 800 × *g* for 10 minutes and the sediment (pellet) was stored at −20 °C and (2) 15 mL was stored as unfractioned native urine at −20 °C.

Cervical scrapes were collected with a Cervex-Brush (Rovers Medical Devices, Oss, The Netherlands) in Thinprep PreservCyt solution (Hologic, Bedwork, MA, US) and stored at 4 °C.

### DNA isolation and bisulfite modification

DNA from urine sediment (15 mL original volume) was isolated using the DNA mini and blood mini kit (Qiagen, Hilden, Germany). DNA from native urine was isolated using the Quick DNA urine kit (Zymo Research, Irvine, CA, US). Following isolation, DNA was eluted in 50 µL elution buffer. DNA from cervical scrapes was isolated using the Nucleo-Spin 96 Tissue kit (Macherey-Nagel, Germany) and Microlab Star robotic system (Hamilton, Germany). All procedures were performed according to the recommendations of the manufacturer.

DNA concentrations were quantified using a NanoDrop 1000 (ThermoFisher Scientific, Waltham, MA, US). Isolated DNA was subjected to bisulfite treatment using the EZ DNA Methylation Kit (Zymo Research, Irvine, CA, US), according to manufacturer’s instructions.

### HrHPV testing

DNA was subjected to hrHPV testing using the HPV-Risk assay (Self-screen B.V., Amsterdam, The Netherlands), a multiplex real-time PCR-based assay for the clinical detection of 15 (probably) hrHPV types, with partial genotyping for HPV16 and HPV18^21^.

### Quantitative methylation specific PCR (qMSP) analysis

Two multiplex qMSPs for each 3 targets (*FAM19A4*, *PHACTR3*, *PRDM14*^19^ and *GHSR*, *SST*, *ZIC1*^18^) and *ACTB* was performed using 50 ng of bisulfite-converted DNA as described before^22^ on an ABI-7500 real-time PCR-system (Applied Biosystems, Waltham, MA, US). *ACTB* was used as a reference gene for quantification and as a quality control. The methylation values of the targets were normalized to *ACTB* using the comparative C_q_ method (2^−ΔCq^ × 100) to obtain C_q_ ratios. Samples with an *ACTB* C_q_ value > 32 were considered to be of insufficient quality and excluded from further analysis. Based on this criterion, 3 native urines (2 controls, 1 cervical cancer), 4 urine sediments (3 controls, 1 cervical cancer), and 2 scrapes from cervical cancer patients were excluded.

### Data analysis

Agreement of hrHPV test results between urine sediment and native urine or cervical scrapes was assessed using Cohen’s kappa statistics^23^. HrHPV agreement was defined as slight (kappa ≤ 0.20), weak (kappa = 0.21–0.40), moderate (kappa = 0.41–0.60), strong (kappa = 0.61–0.80), near-perfect (kappa = 0.81–0.99), and perfect (kappa = 1.000).

The Spearman correlation coefficient *r* was calculated based on log2-transformed C_q_ ratios to assess correlation for each DNA methylation marker between urine sediment and native urine or cervical scrapes.

Differences in DNA methylation levels between patient and control groups were determined using the non-parametric Mann-Whitney U-test. P values < 0.05 were considered statistically significant.

Logistic regression analysis was performed to assess the prediction ability of the 6 DNA methylation markers for detection of cervical cancer. First, the C_q_ ratio of each marker was log2-transformed. Next, univariable logistic regression model was fitted to observe the performance of each marker. The performance of the models was assessed by leave-one-out cross validation (LOOCV), then visualized by the receiver operating characteristics (ROC) curve and quantified by area under the curve (AUC).

All statistical analyses were performed in R open source software, using the pROC package for logistic regression analysis^24^.

### Statement

All experiments and methods were performed in accordance with relevant guidelines and regulations.

## Results

### Comparison of hrHPV and methylation analysis in urine sediment versus native urine

To evaluate which urine component (sediment or native urine) is best suited for hrHPV detection and DNA methylation analysis we analyzed paired urine sediments and native urine from 28 women with cervical cancer. HrHPV was detected in 25 (89%) urine sediments and 24 (86%) native urine samples from cervical cancer patients (Table 1; upper part), with the majority of the hrHPV-positive women being HPV16/18 positive. We found a strong agreement in the detection of hrHPV, overall and at the genotype level, between urine sediments and native urine with a kappa statistic of 0.79 (95% confidence interval (CI) 0.58–1.00).

**Figure 1:**
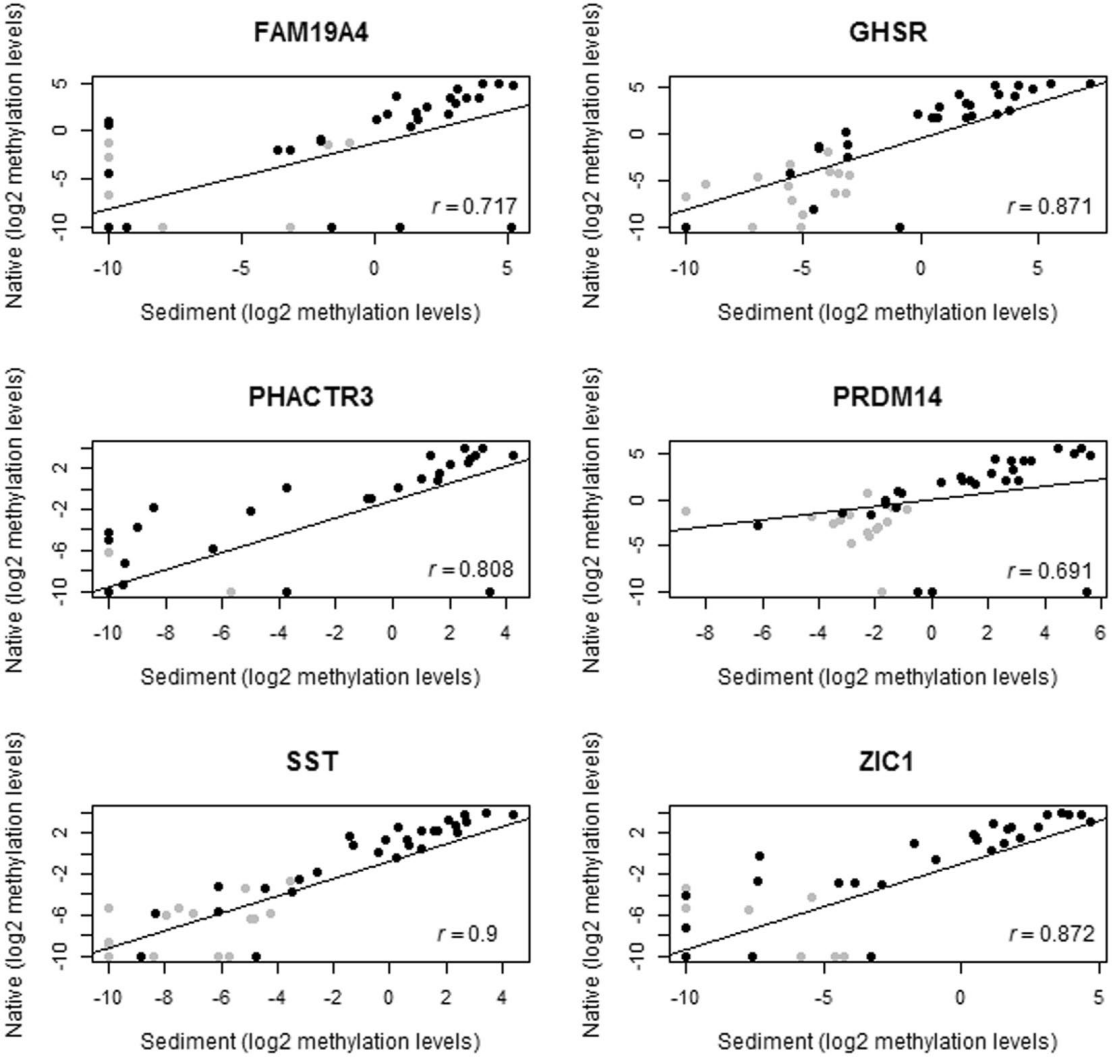
Correlation between paired urine sediment and native urine from 28 women with cervical cancer (black) and 15 controls (gray). Log2 methylation levels of *FAM19A4*, *GHSR*, *PHACTR3*, *PRDM14*, *SST* and *ZIC1* were used. Spearman correlation coefficient (*r*) values are shown.

In addition, a strong correlation was observed between urine sediments and native urine for all 6 DNA methylation markers (*FAM19A4*, *GHSR*, *PHACTR3*, *PRDM14*, *SST*, and *ZIC1*), with correlation coefficient varying from 0.691 (*PRDM14*) to 0.9 (*SST*) (Fig. 1).

Given similar findings and based on practical considerations, such as ease of storage and costs of DNA isolation, we decided to continue with urine sediments.

### hrHPV and DNA methylation detection in paired urine sediments and cervical scrapes

We investigated how hrHPV and DNA methylation test results in urine sediment correlated to cervical scrapes for which we had 38 paired samples available. In the paired samples, hrHPV infection was detected in 31 (82%) urine sediments and 34 (89%) cervical scrapes (Table 1; complete set), resulting in a near-perfect agreement overall and at the genotype level (with a kappa value of 0.85 (95% confidence interval (CI) 0.64–1.00).

**Figure 2:**
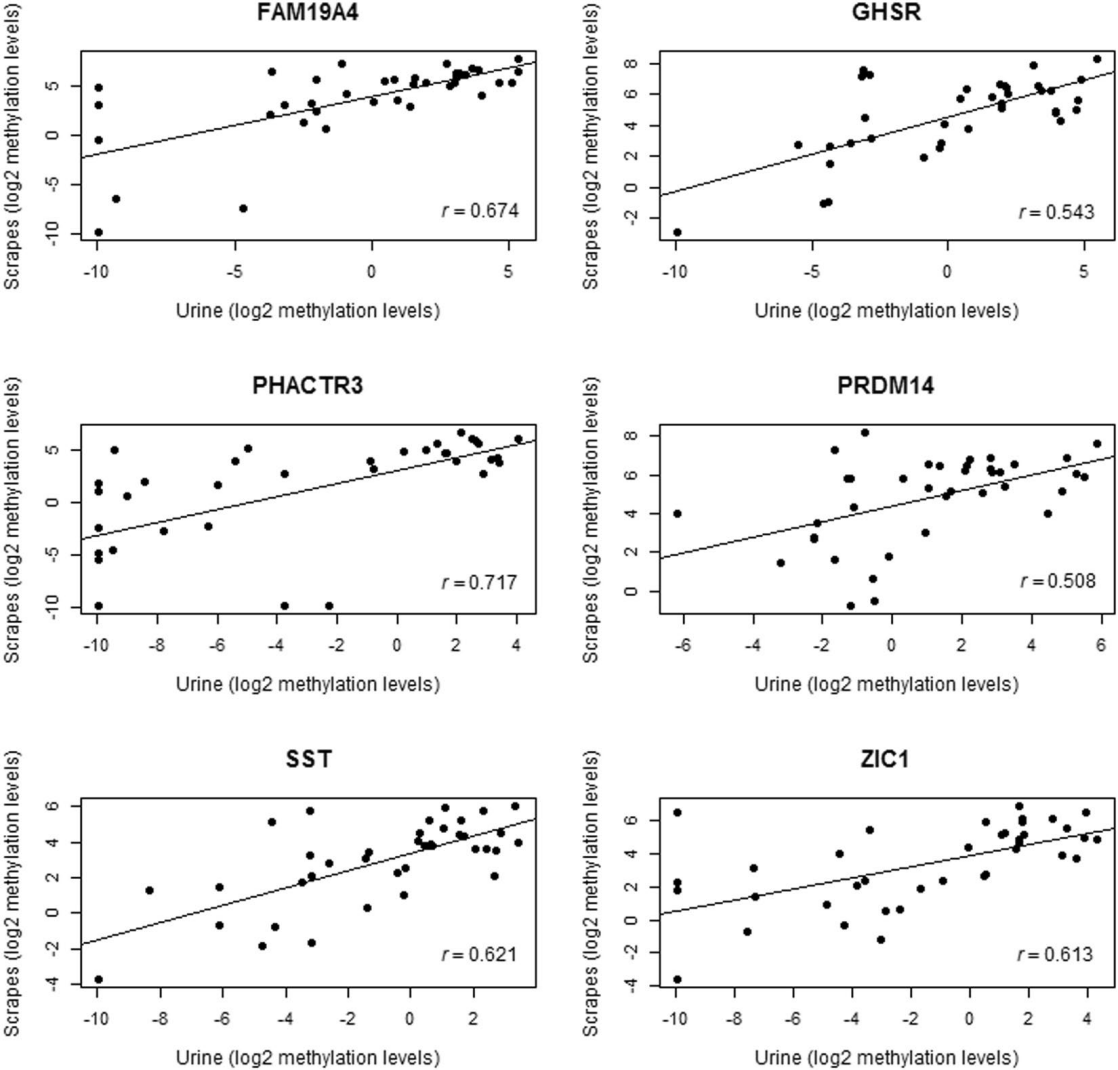
Correlation between paired urine sediment and cervical scrapes from 38 women with cervical cancer. Log2 methylation levels of *FAM19A4*, *GHSR*, *PHACTR3*, *PRDM14*, *SST* and *ZIC1* were used. Spearman correlation coefficient (*r*) values are shown.

For the DNA methylation markers we obtained a moderate to strong correlation of DNA methylation levels between urine sediments and cervical scrapes (correlation coefficient varying from 0.508 (*PRDM14*) to 0.717 (*PHACTR3*); Fig. 2). Notably, the DNA methylation levels in cervical scrapes were considerably higher than those in urine (data not shown).

### Clinical performance of DNA methylation markers in urine sediments

**Figure 3:**
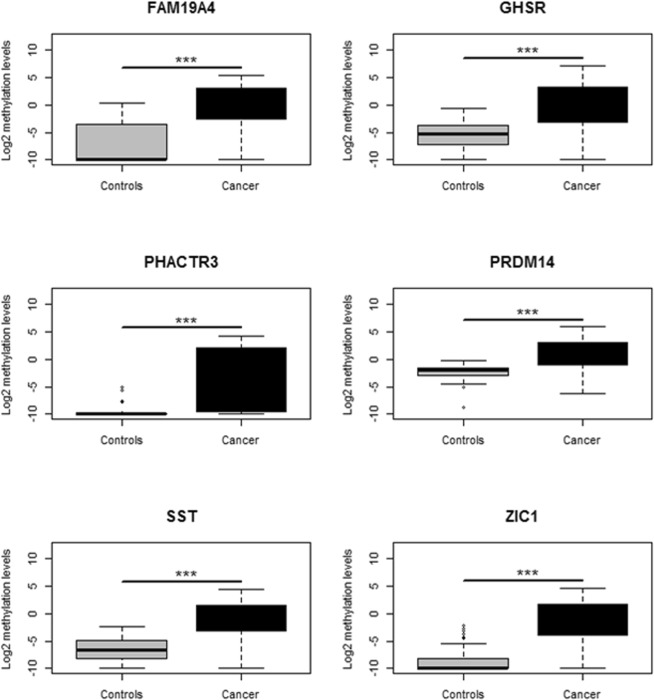
Methylation levels of *FAM19A4*, *GHSR*, *PHACTR3*, *PRDM14*, *SST* and *ZIC1* in urine sediments from 42 cervical cancer patients (black) and 42 controls (gray). Methylation levels were determined by multiplex qMSP and represented by the log2-transformed C_q_ ratios.

Subsequently, we assessed whether the 6 DNA methylation markers can discriminate between urine sediments from women with and without cervical cancer. Comparison of urine sediments from cervical cancer patients to controls revealed a significant increase in DNA methylation levels for all DNA methylation markers in cervical cancer patients (*P* < 0.001; Fig. 3).

**Figure 4:**
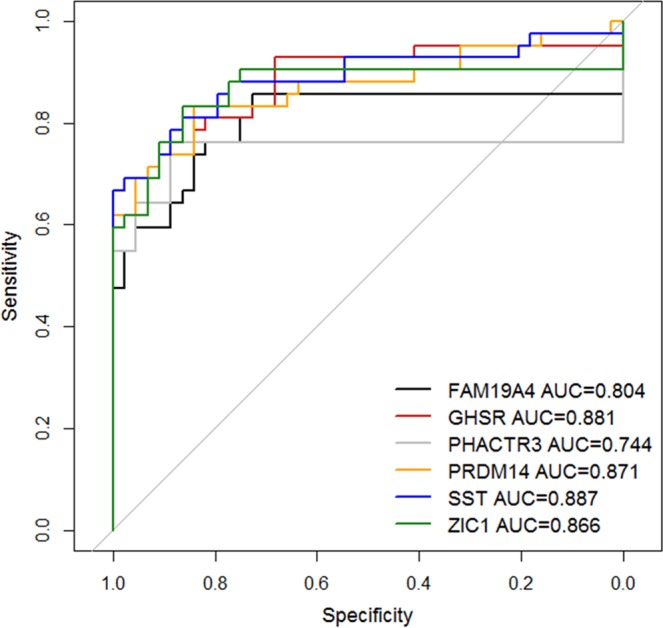
Receiver operating characteristics (ROC) curve and AUC of *FAM19A4* (black), *GHSR* (red), *PHACTR3* (gray), *PRDM14* (orange), *SST* (blue) and *ZIC1* (green) in urine sediments from 42 cervical cancer patients and 44 controls.

Next, we performed univariable logistic regression analysis and LOOCV to assess the performance of each DNA methylation marker in urine sediments. We found that all 6 DNA methylation markers had a high discriminatory power for cervical cancer detection with an AUC varying from 0.744 (*PHACTR3*) to 0.887 (*SST*) (Fig. 4). At the threshold corresponding to 80% specificity in controls, all 6 DNA methylation markers revealed a high sensitivity varying from 76% (*FAM19A4* and *PHACTR3*) to 83% (*PRDM14* and *ZIC1*) for cervical cancer detection in urine sediments. As shown in Table 2 all but one urine sample of cervical cancer patients tested positive for at least one out of 6 DNA methylation markers.

**Table 2:** Overview of logistic regression results in urine sediments from cervical cancer patients.

	1	2	3	4	5	6	7	8	9	10	11	12	13	14	15	16	17	18	19	20	21	22	23	24	25	26	27	28	29	30	31	32	33	34	35	36	37	38	39	40	41	42
FAM19A4	X	X	X		X	X		X		X		X	X	X		X	X	X	X	X	X	X	X	X			X	X	X	X	X	X		X	X	X	X	X		X		
GHSR	X	X	X			X	X	X	X	X	X	X	X	X		X	X	X	X	X	X	X		X		X	X	X	X	X	X	X		X	X	X	X		X	X	X	
PHACTR3	X	X	X		X	X	X	X	X	X	X	X	X	X		X	X	X	X	X	X	X		X			X	X	X	X		X		X	X	X		X		X	X	
PRDM14	X	X	X	X		X	X	X		X	X	X	X	X		X	X	X		X	X	X	X	X		X	X	X	X	X	X	X	X	X	X		X	X	X	X	X	
SST	X	X	X			X	X	X		X	X	X	X	X		X	X	X	X	X	X			X	X	X	X	X	X	X	X	X		X	X	X	X	X	X	X	X	
ZIC1	X	X	X	X	X	X		X		X	X	X	X	X		X	X	X	X	X	X			X	X		X	X	X	X	X	X		X	X	X	X	X	X	X	X	X

Results from the 6 DNA methylation markers (*FAM19A4*, *GHSR*, *PHACTR3*, *PRDM14*, *SST*, and *ZIC1*) are defined as methylation-positive (indicated by X) and methylation-negative (white boxes) at the threshold corresponding to 80% specificity in controls. Numbers indicate cervical cancer patients and correspond to Table 1.

## Discussion

Urine collection has been proposed as a promising alternative for cervical cancer screening when combined with hrHPV testing^12^. This study shows that DNA methylation marker testing is feasible on urine and enables the detection of cervical cancer. Comparison of hrHPV testing in urine sediments, native urines and cervical scrapes yielded a strong to near-perfect agreement (sediments versus native: kappa = 0.79; 95% CI 0.58–1.00, sediments versus scrapes: kappa = 0.85; 95% CI 0.64–1.00). Similarly, analysis of 6 DNA methylation markers (*FAM19A4*, *GHSR*, *PHACTR3*, *PRDM14*, *SST* and *ZIC1*)^18,19^ showed a strong correlation between DNA methylation levels detected in urine sediment versus native urine and versus cervical scrapes. All markers revealed a high discriminatory power for cervical cancer detection in urine sediments. Logistic regression analysis yielded AUCs ranging from 0.744 (*PHACTR3*) to 0.887 (*ZIC1*). This indicates the potential of hrHPV DNA and DNA methylation testing in urine for the detection of cervical cancer.

The near-perfect agreement (kappa = 0.88) of hrHPV detection in paired urine samples and cervical scrapes is in concordance with earlier research, in which agreement of hrHPV detection in urine and cervical scrapes varied from 70% to 96%^25–33^. These studies also indicated the need for optimization and standardization on urine sampling methods, storage conditions, and DNA testing. Of note, in our study 8 out of 42 urine sediments from cervical cancer patients were hrHPV-negative, while for 4 patients the paired cervical scrape was hrHPV-positive. Since higher hrHPV detection rates in cervical samples as compared to urine are commonly observed^34^, this likely explains discrepancies in hrHPV-positivity between urine sediments and cervical scrapes. Testing of the corresponding tissue specimens for the 4 patients that were hrHPV-negative in both urine and cervical samples confirmed a hrHPV-negative test result in three cases (data not shown). Interestingly, all 8 hrHPV-negative urine samples tested methylation-positive for at least 1 DNA methylation marker.

In order to optimize methods, we evaluated detection of hrHPV and DNA methylation markers in urine sediments and native urines and found no differences. The decision to proceed with urine sediments instead of native urine samples was based on practical considerations, such as ease of storage and costs of DNA isolation.

We did not standardize timing of urine collection (first void, midstream or random), as opposed to previous studies^35,36^. Pathak and colleagues (2014) demonstrated that the sensitivity of the hrHPV test could increase when first void urine samples are used^35^. However, results must be interpreted with caution due to heterogeneity in testing methods and results of individual studies. A different definition on “first void” is used in individual studies, as some defined it as the initial stream (correct definition) while others as the first urine sample of the day.

To the best of our knowledge, only three studies compared hrHPV detection in different urine samples in the same population of women. Senkomago and colleagues (2016) found in 27 hrHPV-positive women, no differences in hrHPV detection for first-void, initial stream, and mid-stream urine for sediments^37^. However, Vorsters (2014) found in a pilot study of 10 hrHPV-positive women higher hrHPV DNA detection in first void than mid-stream urine^38^. Leeman and colleagues (2017) reported that physician-taken smears, brush-based self-sampling, morning first-void urine and first-void urine from later during the day showed similar high sensitivity for detection of CIN2+ in their referral population, measured with two different hrHPV tests^39^.

Similar to our hrHPV results, we also showed a strong correlation between DNA methylation levels detected in urine sediment versus native urine (*r* = 0.691 to 0.9) and in urine sediment versus cervical scrapes (*r* = 0.508 to 0.717).

Two previous studies report on DNA methylation analysis in urine for the detection of cervical cancer, but did not compare urine fractions and included other DNA methylation markers^40,41^. Feng and colleagues (2007) found a moderate to high agreement for detection of DNA methylation in urine and cervical scrapes, varying from 49% (*DAPK1*) and 59% (*RARB*) to 80% (*TWIST1*; *CDH13*)^40^, which is consistent with our findings. At least one of the four genes was positive for hypermethylation in 62% of urine from cervical cancer cases, compared with 97% positive for at least one of the six markers in our study. Moreover, in our study, only one out of 42 urine samples from cervical cancer patients was methylation-negative for all six markers.

At the individual gene level all six markers had a high discriminatory power for cervical cancer detection in urine sediments (AUC varying from 0.744 (*PHACTR3*) to 0.887 (*SST*). When it can be confirmed that these DNA methylation markers allow for the detection of clinically relevant precancerous lesions as well, urine-based DNA methylation testing comes into reach for cervical screening. In fact, recent data showed that analysis of a 4-gene methylation classifier (*ZNF516*, *FKBP6*, *INTS1* and *HPV16-L1*) in urine resulted in an AUC of 0.86 when comparing women with CIN1 or no cervical neoplasia (NILM) to CIN2+ lesions^41^.

The advantages of cervical screening using urine over conventional cervical scrapes and self-samples relate to the fact that use of urine is expected to largely increase screening attendance^9,12,35,42^. Furthermore, the use of urine for primary hrHPV-based screening offers an easily manageable manner of sampling^13^.

A study limitation includes the fact that the control urine samples were obtained from women for which we lack knowledge on prior HPV and disease status. Besides the inclusion of better defined controls, e.g. from a regular screening population, further studies including women with cervical precancerous lesions are needed to determine the potential of urine-based cervical screening. Moreover, further standardization of sample collection and optimization of hrHPV testing and DNA methylation marker selection is warranted.

In conclusion, our study demonstrates a good agreement of both urine-based HPV DNA and urine-based DNA methylation analysis with reference cervical samples, and shows the feasibility of DNA methylation testing in urine for the detection of cervical cancer.

## Data Availability

The data generated and analyzed during the current study are available from the corresponding author on reasonable request.
